# Supplement With Calcium or Alendronate Suppresses Osteopenia Due to Long Term Rabeprazole Treatment in Female Mice: Influence on Bone TRAP and Osteopontin Levels

**DOI:** 10.3389/fphar.2020.00583

**Published:** 2020-05-13

**Authors:** Aly A.M. Shaalan, Mohamed El-Sherbiny, Taghrid B. El-Abaseri, Mohamed Z. Shoaeir, Tarek M. Abdel-Aziz, Magda I. Mohamed, Sawsan A. Zaitone, Hala M. F. Mohammad

**Affiliations:** ^1^Department of Histology and Cell Biology, Faculty of Medicine, Suez Canal University, Ismailia, Egypt; ^2^Department of Anatomy, Faculty of Medicine, Jazan University, Jazan, Saudi Arabia; ^3^Department of Human Anatomy and Embryology, Faculty of Medicine, Mansoura University, Mansoura, Egypt; ^4^Department of Anatomy, College of Medicine, Almaarefa University, Riyadh, Saudi Arabia; ^5^Department of Medical Biochemistry and Molecular Biology, Faculty of Medicine, Suez Canal University, Ismailia, Egypt; ^6^Department of Rheumatology and Rehabilitation, Al-Azhar Asyut Faculty of Medicine for Men, Asyut, Egypt; ^7^Department of Physiology, Faculty of Medicine, Suez Canal University, Ismailia, Egypt; ^8^Department of Pharmacology and Toxicology, Faculty of Pharmacy, Suez Canal University, Ismailia, Egypt; ^9^Department of Pharmacology and Toxicology, Faculty of Pharmacy, University of Tabuk, Tabuk, Saudi Arabia; ^10^Department of Clinical Pharmacology, Faculty of Medicine, Suez Canal University, Ismailia, Egypt; ^11^Central Laboratory, Center of Excellence in Molecular and Cellular Medicine (CEMCM), Faculty of Medicine, Suez Canal University, Ismailia, Egypt

**Keywords:** alendronate, calcium, osteopenia, osteopontin, rabeprazole, tartrate resistant acid phosphatase

## Abstract

**Background and Purpose:**

Rabeprazole, a proton pump inhibitor (PPIs) is much endorsed to patients with increased gastric acidity. PPIs were accused to have osteoporotic effects on patients who chronically use them. The point of the current investigation was to decide the impact of rabeprazole on osteoporosis and to explore the modulatory effects of dietary calcium or alendronate on this side effect.

**Methods:**

80 female mice were alienated into four groups maintained for 18 weeks: [1] Vehicle group: given distilled water in 12 ml/kg, P.O. [2] Rabeprazole control group: given rabeprazole in a dose equals 10 mg/kg every 48 h, P.O. [3] Rabeprazole + calcium: given rabeprazole (10 mg/kg every 48 h) along with calcium supplement. [4] Rabeprazole + alendronate: given rabeprazole (10 mg/kg every 48 h) and alendronate (1 mg/kg per week, i.p.). Serum calcium, phosphorus and parathyroid hormone were measured. Both femurs were kept in paraformaldehyde, and then the right one was used for X-ray examination with analysis by Digora software and the left one for histopathological examination (H&E) and immunohistochemical stains for osteopontin and tartrate resistant acid phosphatase (TRAP).

**Results:**

Calcium supplementation or administration of alendronate along with rabeprazole significantly restored the mean bone density as shown by X-ray analysis. Femurs from mice received rabeprazole showed widely separated, thin-walled bone trabeculae and increased number of osteoclasts. Calcium or alendronate with rabeprazole showed thick bone trabeculae without full recovery from rabeprazole induced damage. Adding calcium supplementation to rabeprazole did not affect the histological abnormalities related to osteoclasts meanwhile alendronate produced inactivation of osteoclasts. Both calcium and alendronate decreased the rabeprazole-induced increment in the femur osteopontin level.

**Conclusion:**

Calcium or alendronate can be recommended for female patients on PPI therapy who are at risk of osteopenia.

## Introduction

Proton pump inhibitors (PPIs) are powerful inhibitors of acid production (Eslami et al., 2013). H^+^/K^+^ATPase is the last phase of acid production and PPIs were invented to act on the gastric parietal cells inhibiting this enzyme (Aihara et al., 2003) when systemically absorbed (Savarino et al., 2009). Since their introduction in the 1989, they became one of the supreme prescribed drug classes in the world due to great efficiency in acid-related conditions and its preventive and curative role on gastric ulcerations and gastroesophageal reflux (Pilotto et al., 2006; Nehra et al., 2018).

There was a growing concern regarding the probable over prescription of PPIs (Forgacs and Loganayagam, 2008) and their possible side effects (Parikh and Howden, 2010). Recently, the Food and Drug Administration reported worrying about bigger osteoporosis risk of fractures prompted by long term treatment with PPI (Nehra et al., 2018).

The association between therapy with a PPI and risk of fracture has been studied earlier. Some studies showed rise in spine, hip or total fractures with long term PPI consumption (Kwok et al., 2011; Khalili et al., 2012) while others failed to prove this association (Kaye and Jick, 2008; Yu et al., 2008).

Some physiologic mechanisms are thought to be responsible for PPIs induced osteoporosis including decreased calcium absorption due to hypochlorhydria (Recker, 1985) and/or a direct PPI inhibitory action on osteoclastic H^+/^K^+^ATPase (Sheraly et al., 2009). Studies did not provide a certain conclusion about the exact mechanism for amplified hip fracture risk (Hansen et al., 2010) and the influence of PPIs on bone mineral density (BMD) is still currently under investigation.

The bisphosphonate, alendronate, is a commonly used anti-osteoporotic medication that alleviates the trabecular bone loss induced by gastrectomy (Suzuki et al., 2005). Calcium alendronate inhibits osteoclastic activity and decrease proliferation and differentiation of osteoclasts (Boanini et al., 2015).

To better elucidate the link between PPIs and osteopenia, it is important to explore its biologic probability by investigating its pathologic features. Since osteoporosis and related fractures are found largely in females relative to males. Hence, the current study aimed to examine the negative effect of long term rabeprazole regimen on femur BMD, histopathologic features and osteoclastic activity in female mice. Further, the study evaluated the outcome of using calcium supplementation or pharmacologic treatment with alendronate in reducing osteopenia induced by rabeprazole. This was estimated by X-ray scanning for BMD, histopathological and immunohistochemical means.

## Materials and Methods

### Animals and Their Environment

The present study comprised 80 female Swiss albino mice [body weight equals 18–26 g] were obtained from Moustafa Rashed Company in Giza, Egypt. Mice were grouped and kept in separate polyethylene cages under normal dark/light cycle with balanced standard chow diet and fresh-water supply provided *ad libitum*. The experiment was approved by the Faculty of Medicine research ethics committee, Suez Canal University (#4096).

### Drugs and Chemical Reagents

Rabeprazole and alendronate were kindly gifted by Sigma Pharmaceutical Co. (Quesna City, Menoufya, Egypt). Rabeprazole was dissolved in distilled water for oral administration while alendronate was dissolved in sterile saline solution for I.P. use. Calcium carbonate was purchased from a commercial source (Al-Gomhuria Company for drugs and medicinal supplies, Cairo, Egypt).

### Grouping and Dosing

A couple of experiments were done at different occasions. In each experiment, forty female mice were divided into four groups. The results shown in this article are the average of the two experiments. Treatment with rabeprazole, calcium or alendronate was set as follows:

Group I (Vehicle group): 20 mice were given distilled water (vehicle of rabeprazole) in 12 ml/kg, P.O., for 18 weeks.

Group II (Rabeprazole control group): included 20 mice given 10 mg/kg rabeprazole every 48 h, P.O., for 18 weeks.

Group III (Rabeprazole + calcium): included 20 mice given rabeprazole (10 mg/kg every 48 h) along with dietary calcium carbonate (0.5% w/w mixed thoroughly with chow diet), for 18 weeks (Gorgels et al., 2010).

Group IV (Rabeprazole + alendronate): included 20 mice given rabeprazole (10 mg/kg every 48 h) and alendronate (1 mg/kg/week, i.p.) (Nagai et al., 2007) for 18 weeks.

After completion of the experiment, blood was collected from mice by intracardiac puncture then animals were sacrificed by cervical dislocation. One milliliter of the collected blood samples was subjected to centrifugation to separate the serum. Clear serum samples were stored in Eppendorf tubes and stored at − 80°C. The right femurs were kept in paraformaldehyde and used for X-ray examination. The left femur was excised and kept in paraformaldehyde for histopathological examination.

### Measuring Serum Ionized Calcium, Phosphorus and Parathyroid Hormone

Serum calcium level was quantitatively determined using SPINREACT colorimetric kit (Spinreact, S.A./S.A.U. Ctra. Santa Coloma, Spain). The reaction occurs in alkaline medium and depends on development of colored complex from calcium and o-cresolphtalein. The color strength proportionates to the amount of calcium in the sample (Connerty and Briggs, 1966).

Mouse parathyroid hormone (PTH) ELISA kit from MyBiosource (Cat #MBS704631) was used for determination of PTH in mice sera. The assay kit is a quantitative sandwich ELISA. PTH specific antibodies have been pre-coated onto a microplate. Samples were pipetted into the wells versus standards and the PTH content was bound to the immobilized antibodies.

Phosphorus microplate assay kit from MyBiosource (Cat #MBS8243207) was used for determination of serum phosphorus level. Phosphorus concentration is based on the reaction of phosphorus with ammonium molybdate to form a blue colored product. The color intensity at 620-nm is relative to phosphorus concentration. One hundred microliters of serum samples and 900 μl of the assay buffer were mixed in a microcentrifuge tube and centrifuged at 8,000×*g* at 25°C for 10 min. Then, supernatants were taken into new centrifuge tubes for detection. The reaction buffer and the dye reagent were then added and allowed to react for 10 min and then, the absorbance was read at 620 nm. Concentration of phosphorus in samples was calculated relative to standard concentrations of phosphorus.

### Method for Measurement of Bone Density by Digora Software

Femurs from the experimental groups were kept in formalin. And subjected to X-ray measurement by the digital X-ray unit (FONA XDC type 9319060100, Fona SRL Via Galilei 11 Assao, Italy). Images were then imported into Digora for Windows 2.5 software. Density measurement tool was selected; then, area the distal femur was measured. The software gives minimum, maximum and means density. The computer system uses 0–255 (0 as black and 255 as white). However statistical analysis used the mean density for each animal’s femur.

### Tissue Sampling

Tissue samples (femurs) were obtained from rats after ketamine anesthesia (100 mg/kg, i.p.) and cervical dislocation. Femurs were fixed in 4% paraformaldehyde 24 h at the refrigerator and were then subjected to decalcification in 20% EDTA solution for a couple of hours in a microwave at 50°C and then for 22 h at 4°C. After that, samples were embedded in paraffin wax after dehydration. Four micrometer-thick sections were cut by the aid of a microtome and stained with hematoxylin and eosin (H&E) and immunohistochemistry for osteopontin and tartrate resistant acid phosphatase (TRAP).

### Histopathological Examination of Bone Tissues

First, tissue specimens were examined for arrangement of bone marrow trabecula and intertrabecular spaces in mice. The thickness of trabecula was measured by imageJ software (NIH, USA). Mean thickness for each image was determined at six random points and then the mean value for each group was calculated and compared. The method of measuring trabecular thickness is illustrated in **Supplementary 1**.

Second, H&E-stained bone sections were examined for the pathologic features of osteoclasts e.g. size of the cell, number of nuclei, the appearance of clear zones and length of cytoplasmic processes.

### Immunohistochemical Staining for Tartrate-Resistant Acid Phosphatase and Osteopontin

The first step was blocking of non-specific antigenicity. Then, primary monoclonal antibodies for TRAP (ThermoFisher Scientific, USA) or rabbit polyclonal antibodies for osteopontin (GTX31886, GeneTex, CA, USA) were added to the tissue sections and incubated for an overnight at 4°C. After washing in Tris-buffered saline (TBS), the tissue specimens were incubated with suitable secondary antibodies for 20 min at room temperature. The next step was the incubation with streptavidin for ten minutes. The reaction was detected with 3,3’-diaminobenzidine. Mayer’s hematoxylin was used then for counterstaining.

### Digital Image Analysis (Morphometric Study)

Slides were photographed using Olympus^®^ digital camera installed on Olympus^®^ microscope with 1/2× photo adaptor, using 20× objective. The result images were analyzed on Intel^®^ Core I5^®^ based computer using VideoTest Morphology^®^ software (Russia) with a specific built-in routine for measurement of optical density of immunostained TRAP and to measure area % for immunostained osteopontin (**Supplementary 2**).

### Statistical Analysis for Data

Data were collected and tabulated using Microsoft Excel 2013. Data were demonstrated as mean ± standard deviation (SD). Estimation of statistical significance of the differences was performed by one-way ANOVA and Tukey’s test for multiple comparisons between groups at P-value <0.05. The SPSS program was employed to run these statistical tests.

## Results

In the present study, analysis of serum calcium level by one-way ANOVA test indicated differences among the study group [F(3,20) = 9.69, P-value <0.05]. Multiple comparisons test showed that rabeprazole group showed significantly lower serum calcium level compared to the vehicle treated group (5.5 ± 2.07 vs. 9.68 ± 2.77, **Table 1**). Similarly, mice treated with alendronate along with rabeprazole showed a similar deficiency in serum calcium level compared to the vehicle group (7.35 ± 1 vs. 9.68 ± 2.77). However, mice received calcium supplement along with rabeprazole did not demonstrate a significant difference in serum calcium level in comparison to the vehicle group or the rabeprazole control group; the value was positioned at the middle of both groups means (**Table 1**). Analysis of serum PTH level by one-way ANOVA test indicated differences among the study group [F(3,20) = 4.961, P-value <0.05]. The PTH levels in rabeprazole control group or Rabeprazole + Alendronate group was significantly higher than the vehicle group (43.98 ± 19.23 and 41.48 ± 9.25 vs. 18.69 ± 4.19, P-value <0.05, **Table 1**). Meanwhile, rabeprazole + calcium group did not demonstrate a significant change in PTH in comparison to the vehicle group (**Table 1**). In the present study, analysis of serum phosphorus level by one-way ANOVA test indicated differences among the study groups [F(3,20) = 2.368, P <1.01]. No further analysis was performed.

**Table 1:** Effect treatment with alendronate or calcium supplement on serum levels of PTH, calcium and phosphorus in mice treated with rabeprazole.

Groups	PTH (pg/ml)	Calcium (U/L)	Phosphorus (mmol/L)
**Vehicle**	18.69 ± 4.19	9.68 ± 2.77	9.92 ± 1.21
**Rabeprazole**	43.98 ± 19.23*	5.5 ± 2.07*	7.98 ± 1.08
**Rabeprazole + calcium**	33.9 ± 12.41	7.35 ± 1	9.13 ± 1.11
**Rabeprazole + alendronate**	41.48 ± 9.25*	6.34 ± 0.74*	9.02 ± 1.37

PTH, parathyroid hormone. Data are expressed as mean ± SD and analyzed using one-way ANOVA followed by Tukey’s post-hoc test. *Significantly different from normal group at P < 0.05.

**Figure 1:**
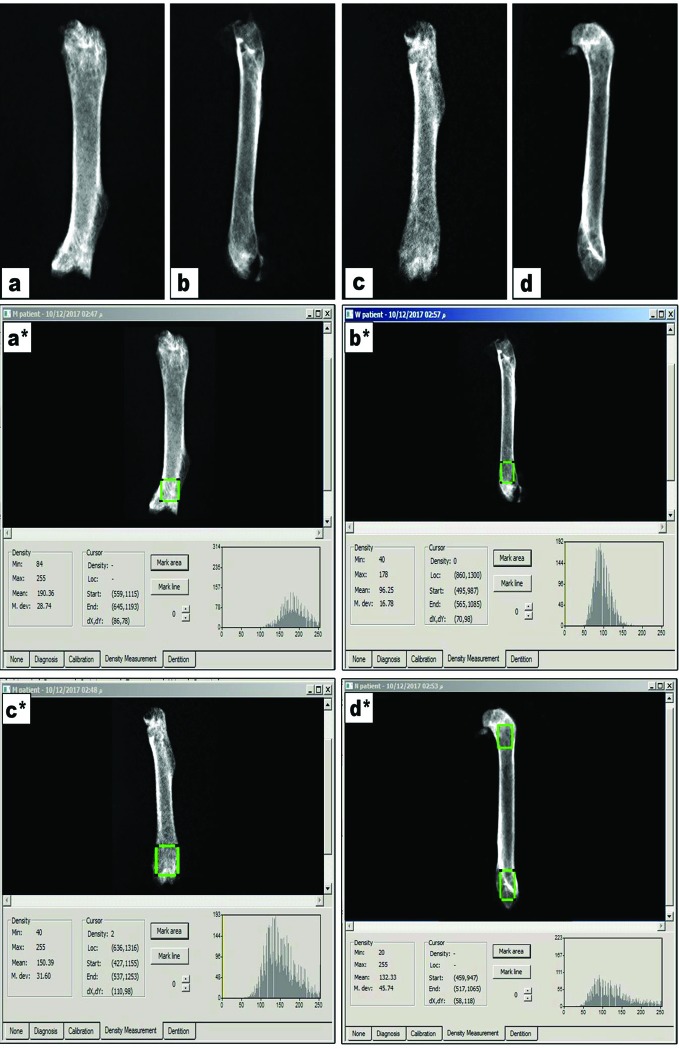
X-ray images for femur bones and radiographic images measured on Digora software. **(A–D)** X-ray images for vehicle, rabeprazole control, rabeprazole + Calcium and rabeprazole + Alendronate, respectively. (A*–D*) Radiographic images measured on Digora software reveal bone density analysis of distal part of the mouse femur marked as square by Digora System, showing the minimum, maximum and the mean density profiles for each image.

**Figure 2:**
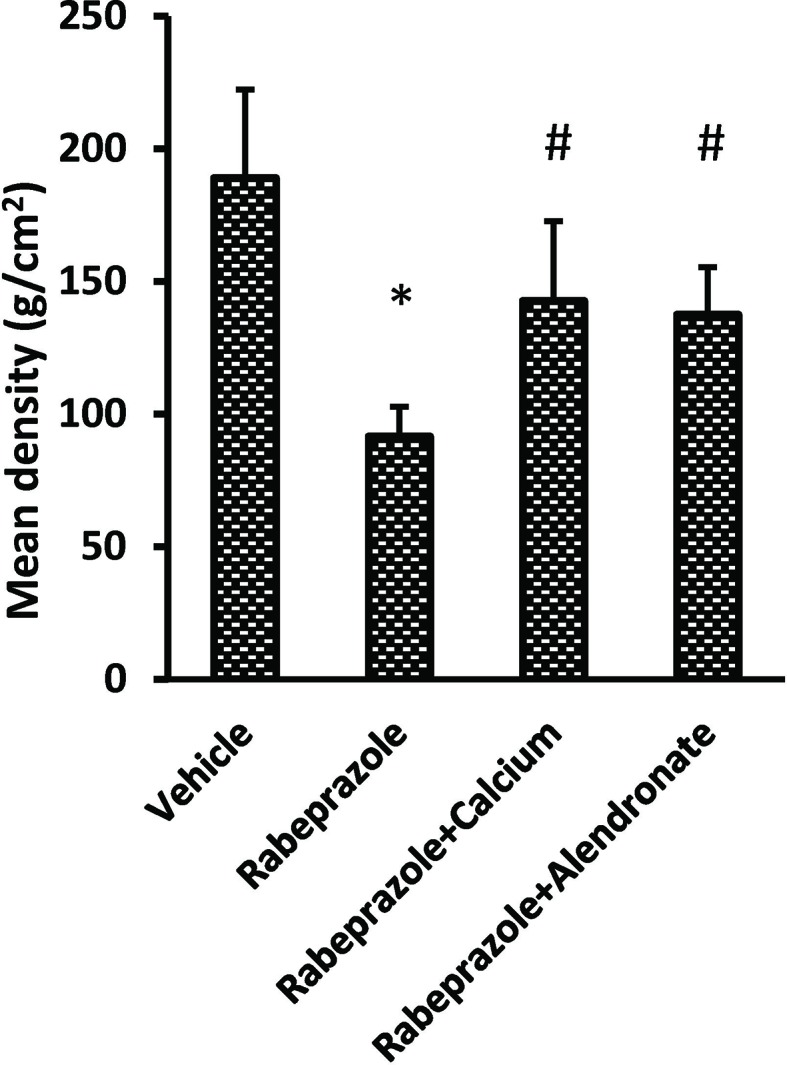
Effect of alendronate or calcium supplement on femur density in mice under long term rabeprazole treatment. Column chart represents the mean bone density. *Comparison to vehicle group, ^#^Comparison to rabeprazole group at P-value <0.05.

**Figure 1** demonstrates X-ray pictures for mice femurs from the different groups (A–D). Measuring X-ray densitometry was done by Digroa software gave the value of maximum, minimum and mean BMD (Figures 1A*–D*). Analysis of BMD results indicated significant differences among the study groups [F(3,20) = 9.262, P-value <0.05]. A significant reduction was observed in mean value of BMD in rabeprazole group in comparison to the vehicle group. However, calcium supplementation or administration of alendronate along with rabeprazole significantly restored the mean BMD (P-value <0.05, **Figure 2**).

**Figure 3:**
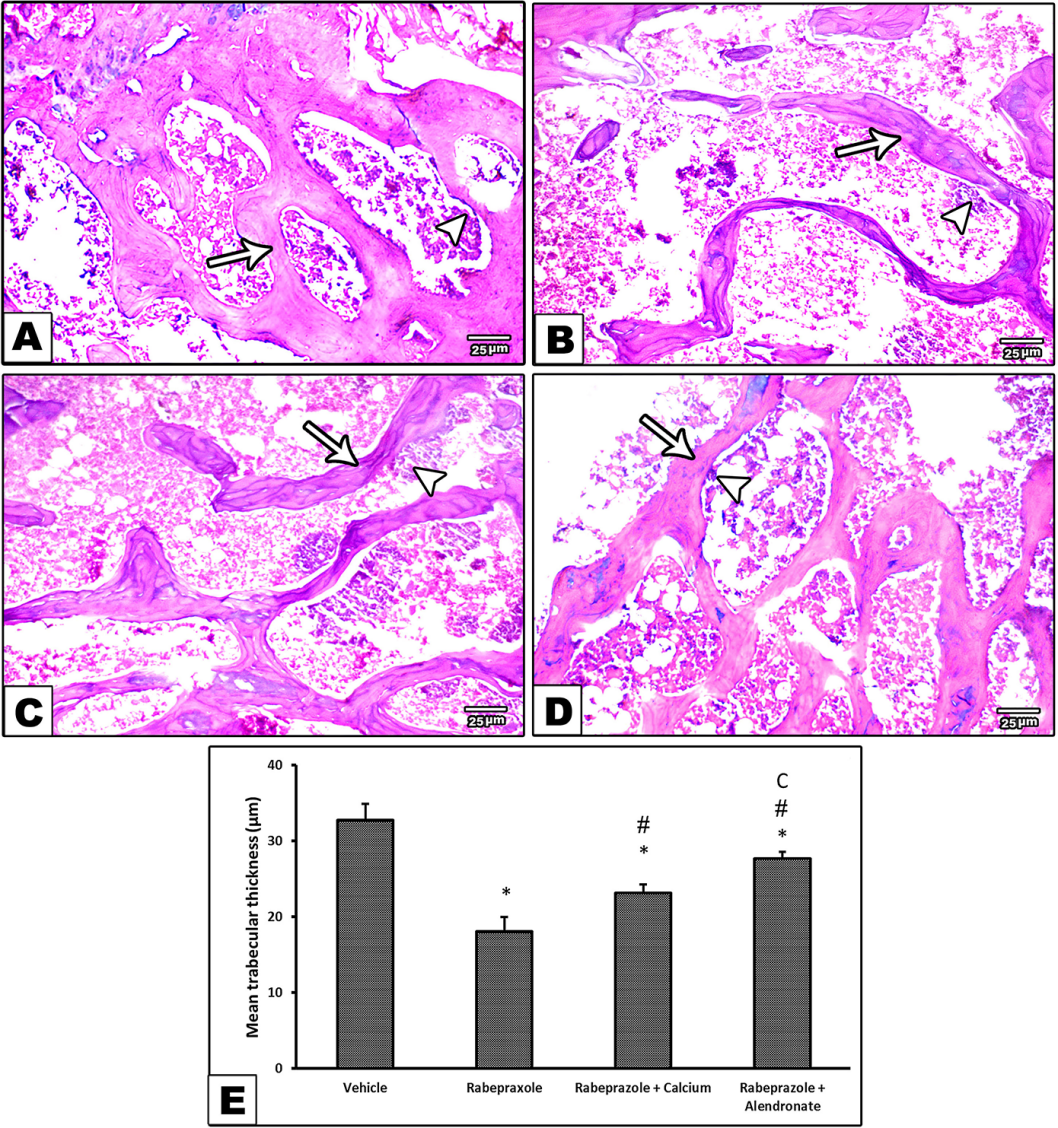
Effect of alendronate or calcium supplement, on the bone trabecula and intertrabecular spaces in mice under long term rabeprazole treatment. Photomicrographs for bone sections stained with hematoxylin and eosin. **(A)** Vehicle group showing normal bone tissue with a parallel arrangement of bone trabeculae (arrow), and an arrowhead pointing to osteoclasts, **(B)** Rabeprazole group showing widely separated, thin-walled bone trabeculae (arrow) with widened inter trabecular spaces containing bone marrow element and apparently increase in the number of osteoclasts (Arrowhead), **(C, D)** Rabeprazole+Calcium group and Rabeprazole+Alendronat group showing thick bone trabeculae (arrows). Trabecular thickness was measured in random 6 points and averaged for each image. Also, arrowheads pointing to osteoclasts. **(E)** Column chart demonstrating the mean trabecular thickness (µm) in the experimental groups. Data are mean±SD, *Comparison to the vehicle, ^#^Comparison to rabeprazole group, ^C^Comparison to rabeprazole+calcium group, at P-value <0.05.

Hematoxylin and eosin staining was investigated for two criteria. First, microscopic examination of bone marrow (BM) trabecula and intertrabecular spaces is shown in **Figure 3**. Sections from femurs from the vehicle group showed normal bone tissue with parallel arrangement of bone trabeculae (**Figure 3A**) whereas, femurs from mice received rabeprazole showed widely separated, thin-walled bone trabeculae with widened inter trabecular spaces containing bone marrow element and increased number of osteoclasts (**Figure 3B**). Meanwhile, mice received calcium or alendronate with rabeprazole showed thick bone trabeculae (**Figures 3C, D**). Further, **Figure 3E** is a column chart showing the mean trabecular thickness in the study groups. One-way ANOVA indicated a significant difference among the study groups [F(3,20) = 88.17, P-value <0.05]. The rabeprazole group showed significant decline in trabecular thickness compared to the vehicle group (18.07 ± 1.89 versus 32.67 ± 2.21, P-value <0.05, **Figure 3E**). Calcium supplementation or alendronate therapy partially restored trabecular thickness compared to the rabeprazole control group 23.13 ± 1.14 and 27.67 ± 0.85 versus 18.07 ± 1.89, P-value <0.05, **Figure 3E**).

**Figure 4:**
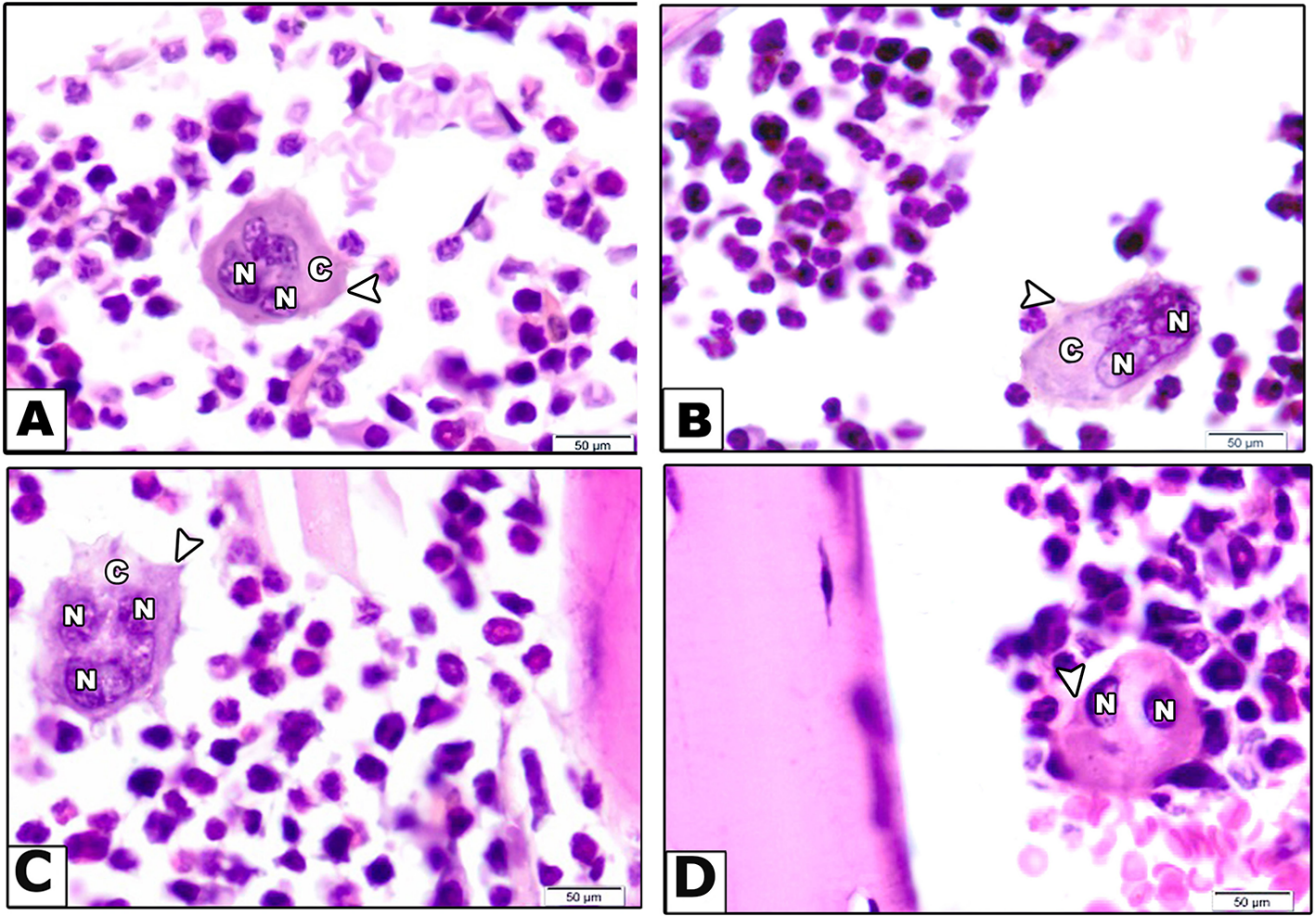
Effect of alendronate or calcium supplement on osteoclast morphology in mice under long term rabeprazole treatment. Photomicrographs for bone sections stained with hematoxylin and eosin in different experimental groups. **(A)** Vehicle group showing bone marrow containing osteoclast as a large cell has multiple nuclei (N), clear zone (C) and short cytoplasmic processes (arrow head). **(B)** Rabeprazole group showing bone marrow containing osteoclast as a large cell has multiple nuclei (N), prominent clear zone (C) and long cytoplasmic processes (arrow head). **(C)** Rabeprazole + Calcium group showing bone marrow containing osteoclast as a large cell has multiple nuclei (N), prominent clear zone (C) and long cytoplasmic processes (arrow head). **(D)** Rabeprazole + Alendronate group showing bone marrow containing quiescent osteoclast as a large cell has two inactive nuclei (N), and short cytoplasmic processes (arrowhead).

Second, examination of H&E sections for osteoclastic activity indicated that vehicle treated mice showed osteoclasts as a large cell has multiple nuclei, clear zone and short cytoplasmic processes (**Figure 4A**). However, rabeprazole group showed osteoclasts as large cells with multiple nuclei, prominent clear zone but with long cytoplasmic processes (**Figure 4B**). Calcium supplementation along with rabeprazole treatment did not change the cytological features of osteoclasts compared to the rabeprazole control group (**Figure 4C**). Differently, mice received alendronate injections along with rabeprazole showed bone marrow containing quiescent osteoclast as a large cell has two inactive nuclei, and short cytoplasmic processes (**Figure 4D**).

**Figure 5:**
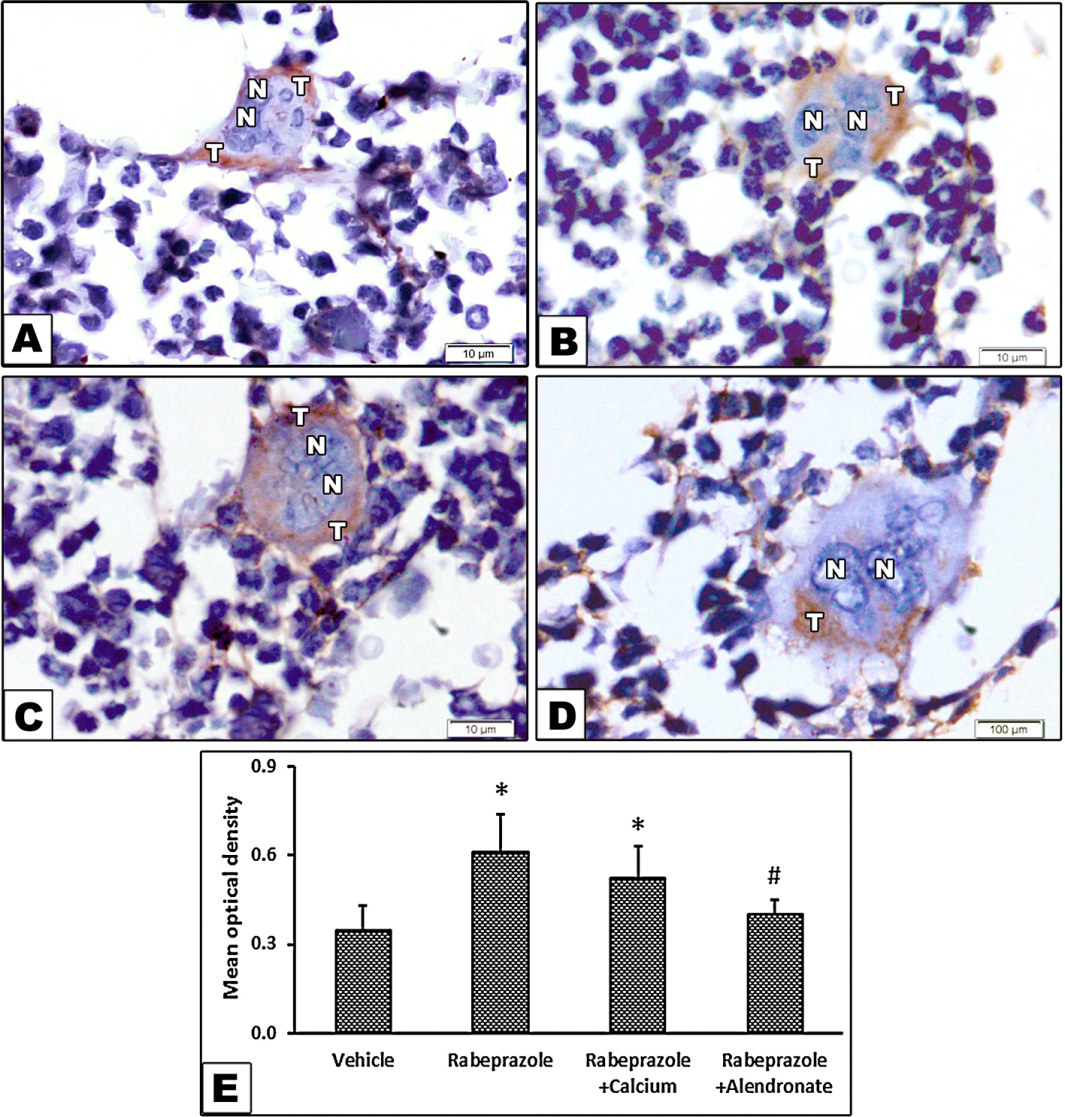
Effect of alendronate or calcium supplement, on the immunostaining for tartrate resistant acid phosphatase in osteoclasts in mice under long term rabeprazole treatment. Photomicrographs for bone sections immunostained for TRAP. **(A)** Vehicle group showing bone marrow containing osteoclast as a large cell has multiple nuclei (N), and cytoplasmic brown stained areas demonstrating the immunostaining of acid phosphatase (T), **(B)** Rabeprazole group showing bone marrow containing osteoclast as a large cell has multiple nuclei (N), and cytoplasmic dark brown stained areas demonstrating the immunostaining of acid phosphatase (T), **(C)** Rabeprazole + Calcium group showing bone marrow containing osteoclast as a large cell has multiple nuclei (N), and cytoplasmic dark brown stained areas demonstrating the immunostaining of acid phosphatase (T). **(D)** Rabeprazole + Alendronate group showing bone marrow containing osteoclast as a large cell has multiple nuclei (N), and cytoplasmic brown stained areas demonstrating the immunostaining of acid phosphatase (T). **(E)** Column chart for optical density of immunostained TRAP in the experimental groups. *Comparison to vehicle group, ^#^Comparison to rabeprazole group at P-value <0.05.

**Figure 5A** shows immunohistochemical staining for TRAP as a biomarker for osteoclastic activity. Image (**A**) from the vehicle group shows bone marrow (BM) containing osteoclast as a large cell has multiple nuclei and cytoplasmic staining of TRAP. Image from rabeprazole group shows cytoplasmic dark brown stained areas (**Figure 5B**). Image from Rabeprazole + Calcium group shows cytoplasmic dark brown staining for TRAP (**Figure 5C**). Image from Rabeprazole + Alendronate group shows cytoplasmic immunostaining for TRAP (**Figure 5D**). Measuring the optical density for TRAP staining indicated differences among the study groups [F(3,20) = 9.62, P-value <0.05]. Higher density was detected in rabeprazole group compared to the vehicle group. Meanwhile, calcium supplementation or administration of alendronate significantly decreased the density of TRAP staining compared to the rabeprazole group (P-value <0.05, **Figure 5E**).

**Figure 6:**
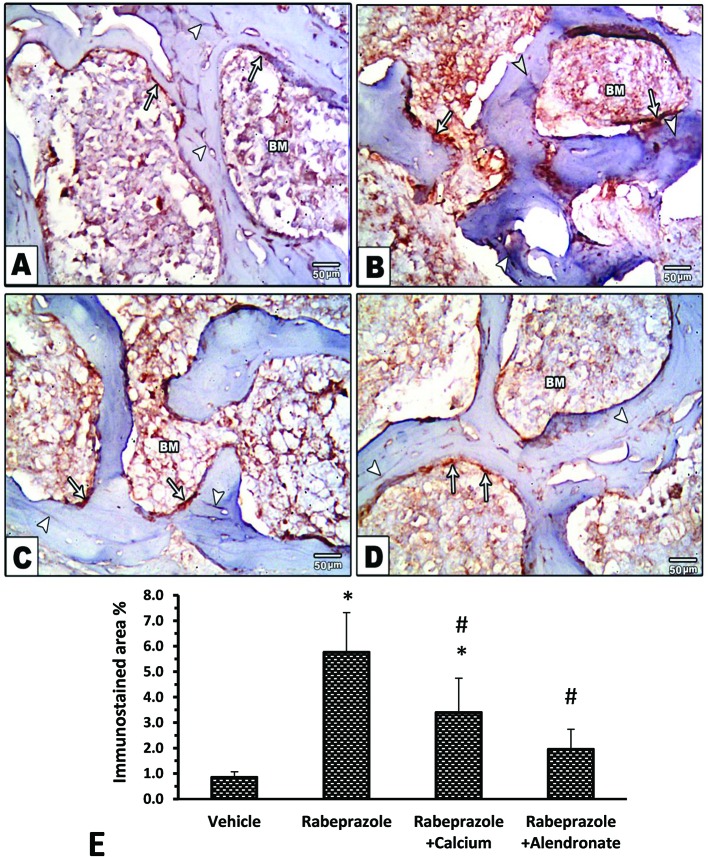
Effect of alendronate or calcium supplement on immunostaining for osteopontin in mice under long term rabeprazole treatment. Photomicrographs for bone marrow sections immunostained for osteopontin. **(A)** Vehicle group showing, bone marrow (BM), endosteum of bone (Arrows) and brown stained lines demonstrating osteopontin in bone (Arrow heads), **(B)** Rabeprazole group showing bone marrow (BM), endosteum of bone (Arrows) and darkly brown stained lines demonstrating osteopontin in bone (Arrow heads), **(C**, **D)** Rabeprazole + Calcium and Rabeprazole + Alendronate groups showing bone marrow (BM), endosteum of bone (Arrows) and darkly brown stained lines demonstrating osteopontin in bone (Arrow heads). **(E)** Column chart for area % of immunostained osteopontin. Data are mean ± SD *Comparison to vehicle group, ^#^Comparison to rabeprazole group at P-value <0.05.

**Figures 6A–D** show images for osteopontin immunostaining in the experimental groups. Image from the vehicle group shows bone marrow (BM), endosteum of bone and brown stained lines demonstrating osteopontin in bone. Image from rabeprazole group shows BM, endosteum of bone and dark brown lines stained for osteopontin (**Figure 6B**). Image from Rabeprazole + Calcium group shows BM, endosteum of bone and dark brown stained lines demonstrating osteopontin in bone (**Figure 6C**). Image from Rabeprazole + Alendronate group shows BM, endosteum of bone and brown stained lines demonstrating osteopontin in bone (**Figure 6D**). Statistical analysis by ANOVA [F(3,20) = 25.61, P-value <0.05] highlighted differences among the study groups. In addition, a significant increase in area of osteopontin immunostaining was found in rabeprazole group compared to the vehicle group and significant declines in rabeprazole + calcium and rabeprazole + alendronate group (P-value <0.05, **Figure 6E**).

## Discussion

### Effect of Rabeprazole on Bone

PPIs are being overprescribed in many health care facilities (Pham et al., 2006) and sometimes for inappropriate indications (Ramirez et al., 2010). The wide consumption of PPIs and reported risk of osteoporosis directed us to test their long term effect in mice and to find out the usefulness of calcium supplement or alendronate therapy in alleviating the skeletal adverse effect of rabeprazole. In the current study, an 18-week rabeprazole course led to a significant decline in BMD and decreased serum calcium level and produced secondary hyperparathyroidism in female mice.

The association between PPIs and the risk of fracture was examined in many studies with variable outcomes (Liu et al., 2019). The first suggested theory described decreased calcium absorption. In agreement, it has been demonstrated that *in vitro* dissolution of calcium carbonate is dependent largely on the pH. Dissolution and possibly absorbable calcium becomes slow and declines with rising pH (Hotta et al., 1999). Furthermore, acid suppressive effect of PPIs was reported to lessen the *in vivo* calcium absorption in elderly fasting women (O’Connell et al., 2005).

In the second suggested theory, PPIs are proposed to weaken osteoclastic activity which is dependent of H^+^/K^+^ATPase, the pump found in the parietal cells (Mizunashi et al., 1993). The two mechanisms could hypothetically result in impaired strength of bones, osteoporosis and bone fractures. The different studies published have not been able to confirm which machinery is crucial for an amplified risk of osteoporosis (Hansen et al., 2010) and still the effects of PPIs on BMI need additional examination. In the current study, we tried to find an explanation for PPI-induced osteopenia.

Consistent with the previous concerns, in our study, X-ray densitometry displayed a significant decline in BMD in mice received long-term rabeprazole therapy. Further, histopathologic examination revealed widely separated, thin-walled bone trabeculae with widened inter trabecular spaces and increased number of osteoclasts. In agreement, one study declared that the use of gastric acid inhibitors (PPIs/H2 antagonists) is linked to a bigger risk of fractures to the hip bone. This finding was only confirmed in persons with—at least—another risk factor predisposing to hip fractures. Hence, inhibition of gastric acid might be linked to risk of fractures in persons who are already at risk for osteoporosis (Corley et al., 2010).

Another study demonstrated a link between PPIs use and hip fracture. The mechanism for the development of hip fracture was unidentified (Targownik et al., 2010). A different study by Yamasaki et al. highlighted that rabeprazole ameliorates BMD 22 weeks after total gastrectomy in male rats without improving bone strength. They concluded that rabeprazole controls osteoclastic bone resorption, similar to the action of bisphosphonates (Yamasaki et al., 2016).

A large population-based Canadian cohort studied the relationship between five or ten years of PPI use and BMD. PPI users acquired less BMD at baseline than non-users but did not show significantly enhanced decline in BMD after five or ten year follow-up period (Targownik et al., 2012). Reimer (2013) suggested that use of PPIs do not associate with enhanced loss of BMD or osteoporosis which is considered as the principal biologic description for elevated fracture risk. They concluded that the worry about fractures of the hip bone and osteoporosis should not avert the indication of long-term PPIs (Reimer, 2013). Differently, a cross sectional and longitudinal investigation of data base of 5-year BMD did not prove a relationship between PPIs and development of osteoporosis (Targownik et al., 2010). Different types of PPIs or species differences may account for this difference.

In the current study, mice treated with rabeprazole showed changes in immunohistochemical staining for TRAP and osteopontin. Greater TRAP immunostaining in their bones compared to the vehicle group; this indicates greater osteoclastic activity Similarly, *in vitro*, the PPIs, lansoprazole, omeprazole and esomeprazole, have shown effects on precursor cells of human osteoclasts sequestered from peripheral blood as well as precursors of osteoblasts (human mesenchymal stem cells). Cell cultures also demonstrated decreased cellular density of osteoclast to osteoblast cells (Costa-Rodrigues et al., 2013).

Osteopontin is an extracellular matrix protein that is expressed in bone cells such as osteoblast and osteocytes and associated with bone turnover and BMD (Wei et al., 2016), it has been implicated in bone remodeling by activating the resorption process (Fodor et al., 2013). Experimental studies showed that mice deficient in osteopontin acquired resistance to ovariectomy-prompted bone resorption. Authors proposed that osteopontin is crucial in women postmenopausal osteoporosis and that approaches trying to diminish the action of osteopontin may show effectiveness in overcoming osteoporosis (Yoshitake et al., 1999). Similarly, the presence of osteopontin was necessary for the impact of mechanical stress upon bone. Evidence came from osteopontin‐null (OPN−/−) mice which did not demonstrate bone resorption enhancement or bone formation suppression when they were exposed to tail suspension test for 4 weeks (Ishijima et al., 2002).

In human studies, serum osteopontin level was in a negative correlation to BMD measured by dual energy X-ray absorptiometry and positive correlation with bone turnover in 362 Chinese postmenopausal women (Wei et al., 2016). Another study examined the relationship between osteopontin, BMD and bone turnover markers and osteoporotic vertebral fractures in 214 postmenopausal women. Authors found elevated osteopontin level is associated with decreased BMD, increased levels of bone turnover markers and osteoporotic vertebral fractures (Fodor et al., 2013).

### Protection by Calcium or Alendronate

In the current study, Ca^2+^ supplementation given along with rabeprazole significantly enhanced the mean BMD. These findings support the first hypothesis declaring that rabeprazole affects calcium absorption and hence deposition.

In one clinical study, Ca^2+^ supplement is efficacious in balancing calcium homeostasis in gastrectomized individuals. Authors analyzed the influence of Ca^2+^supplementation on integrity of the skeletal system in cases with lessened gastric acidification. Undecalcified biopsies taken from bones of 26 gastrectomized people were evaluated by histologic means. Biopsies from these gastrectomized people showed considerably improved indices for osteoid, osteoclast and osteoblast and fibroosteoclasia in addition to diminished distribution for Ca^2+^ in the mineralized matrix of bones in comparison with healthy individuals. Gastrectomy was linked to extensive osteomalacia, marrow fibrosis and reduced distribution of calcium inside the mineralized bone matrix. Similar to the clinical study, Ca carbonate supplementation increased BMD in gastrectomized individuals and restored calcium/skeletal homoeostasis in an animal model of achlorhydria (Krause et al., 2015). Authors concluded that gastrectomy or hypochlorhydria due to long-term PPI therapy are associated with increased fracture risk because of intestinal Ca malabsorption.

Similar to calcium, administration of alendronate along with rabeprazole enhanced the mean BMD and decreased TRAP staining. It is not surprising as bisphosphonates are known to be the present standard for preventing and treating of glucocorticoid-induced osteoporosis (Saag et al., 2007) and postmenopausal osteoporosis in females.

### Significance of Blood Tests

In the current study, hypocalcemia and hyperparathyroidism has been developed secondary to rabeprazole treatment. Secondary hyperparathyroidism leads to normalization of serum calcium in most patients *via* increasing calcium reabsorption from renal tubules and enhanced production of 1,25 dihydroxyvitamin D, which subsequently boosts calcium absorption (Chesnut and Harris, 1993). Patients with dysfunction in the parathyroid gland or impairment of renal function may not be capable to alleviate hypocalcemia, which in turn may develop to be symptomatic (Papapetrou, 2009).

In the current study, serum calcium level was found decreased in mice group received alendronate concomitantly with rabeprazole. Similarly, one study declared that hypocalcemia induced by the bisphosphonate treatment may be intense and persistent and normalization of serum calcium may take up to a couple of months (Gulley et al., 2007). One study reported a postmenopausal female patient with a history of primary hypoparathyroidism displayed intense symptomatic hypocalcemia postadministration of intravenous zoledronic acid for treating her osteoporosis. Further, her serum phosphorus level was also found below the normal level. Therefore, augmented osteoblastic action leading to avid calcium uptake was assumed as a probable mechanism for hypocalcemia provoked by zoledronic acid (Al Elq, 2013).

Regarding human indications, intravenous bisphosphonates are extensively used for managing solid carcinomas, metastatic bone disease and hypercalcemia related to malignancies (Al Elq, 2013). Indeed, elevated osteoblastic function results in increased uptake of calcium and has been assumed as a probable mechanism mediates hypocalcemia prompted by zoledronic acid (Ho and Sundar, 2012). This drug effect is frequently tailed by a compensatory rise in PTH.

Anti-resorptive drugs reduce bone resorption however; PTH boosts bone strength mainly by stimulating the process of bone formation. One randomized double-blind clinical study tested the simultaneous administration of PTH and alendronate would enhance BMD more than the per se drug administration. More than two hundreds low BMD postmenopausal women at the hip or spine were included. No evidence was found on synergy between PTH and alendronate (Black et al., 2003). Authors highlighted that, as expected, they found low serum calcium concentration among the women on alendronate. Meanwhile women treated with PTH showed a rise in mean serum calcium level at 1 and 3 months, which returned to the base line estimate one year later (Black et al., 2003).

One of the limitations of the current study is that we did not measure gastric acid production. We cannot rule out whether calcium carbonate used in the study suppresses the function of rabeprazole or not. Hence, further studies are warranted to assess this point.

This study described the outcome of utilizing alendronate or calcium in combination with rabeprazole however; the detailed mechanisms for rabeprazole-induced osteopenia need to be further elucidated.

## Conclusion

Overall, the current *in vivo* experiment demonstrated that calcium supplementation or alendronate could partially protect against the adverse effect of long term rabeprazole regimen in female mice. The protective effect included partially enhanced BMD and downregulation of bone TRAP and osteopontin levels without full recovery from damage induced by rabeprazole.

Hence, we found it acceptable to recommend conduction of human trials using them for female patients on long PPI therapy who are at risk of osteopenia or osteoporosis. The two options, calcium or alendronate, are available; however calcium is a safer option for long term use given the known adverse effects of alendronate may be anxious for patients.

## Data Availability Statement

All datasets generated for this study are included in the article/**Supplementary Material**.

## Ethics Statement

The animal study was reviewed and approved by Research Ethics Committee at Faculty of Medicine, Suez Canal University, Ismailia, Egypt.

## Author Contributions

Conceptualization: MM, SZ, HM. Resources: all authors. Methodology: all authors. X-ray examination and analysis: ME, MS, TA, HM. Graphic presentation and data curation: all authors. Evaluation: all authors. Writing the manuscript: all authors. Revising and approving the final form: all authors.

## Funding

Authors wish to acknowledge Almaarefa University for the financial support for the publication fees of this article.

## Conflict of Interest

The authors declare that the research was conducted in the absence of any commercial or financial relationships that could be construed as a potential conflict of interest.
