# Supplementary figures and images for: Supplement With Calcium or Alendronate Suppresses Osteopenia Due to Long Term Rabeprazole Treatment in Female Mice: Influence on Bone TRAP and Osteopontin Levels
Source: Front Pharmacol. 2020 May 13;11:583. doi: 10.3389/fphar.2020.00583 (PMC7237708; doi:10.3389/fphar.2020.00583)

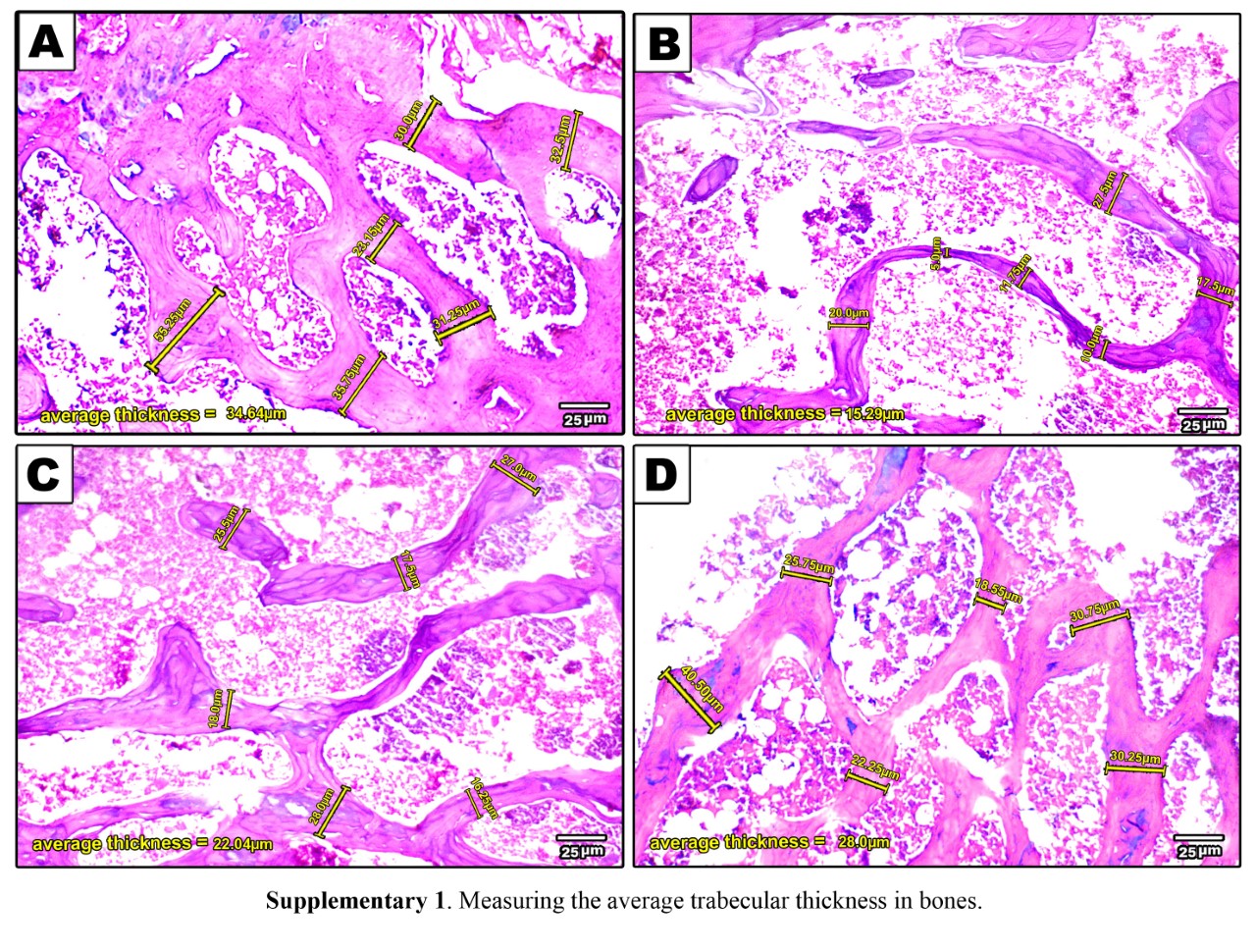

Supplement: Supplementary file 1 [file Image_1.jpg]

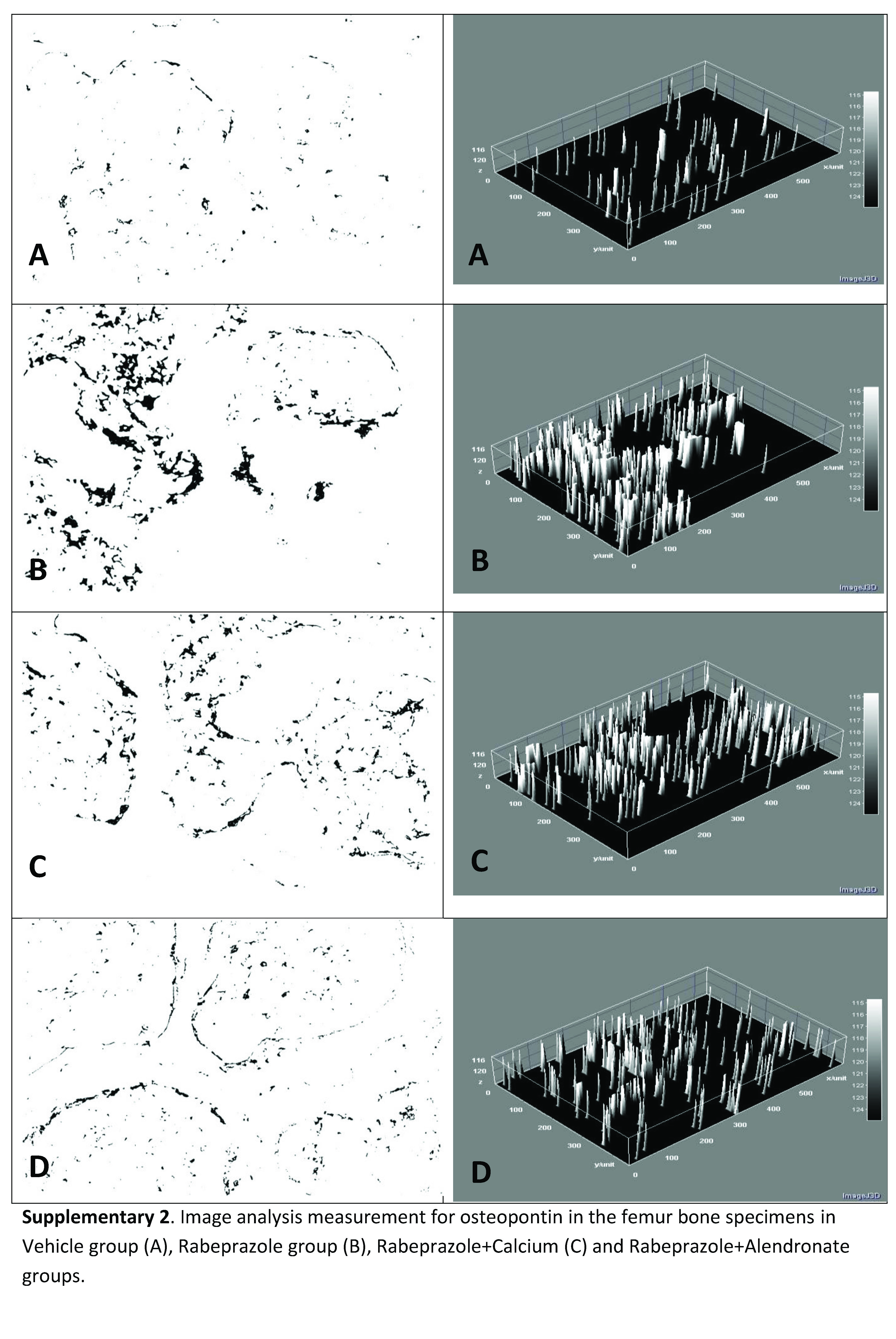

Supplement: Supplementary file 2 [file Image_2.jpeg]
